# Differential Water Mite Parasitism, Phenoloxidase Activity, and Resistance to Mites Are Unrelated across Pairs of Related Damselfly Species

**DOI:** 10.1371/journal.pone.0115539

**Published:** 2015-02-06

**Authors:** Julia J. Mlynarek, Arne Iserbyt, Laura Nagel, Mark R. Forbes

**Affiliations:** 1 Biology Department, Carleton University, Ottawa, ON, Canada; 2 Department of Biology, University of Antwerp, Evolutionary Ecology Group, Groenenborgerlaan 171, Antwerp, B-2020, Belgium; 3 Department of Biology, University of Antwerp, Ethology Group, Universiteitsplein 1, Wilrijk, B-2610, Belgium; 4 Biology Department, Queen’s University, Kingston, ON, Canada; Uppsala University, SWEDEN

## Abstract

Related host species often demonstrate differences in prevalence and/or intensity of infection by particular parasite species, as well as different levels of resistance to those parasites. The mechanisms underlying this interspecific variation in parasitism and resistance expression are not well understood. Surprisingly, few researchers have assessed relations between actual levels of parasitism and resistance to parasites seen in nature across multiple host species. The main goal of this study was to determine whether interspecific variation in resistance against ectoparasitic larval water mites either was *predictive of* interspecific variation in parasitism for ten closely related species of damselflies (grouped into five “species pairs”), or was *predicted by* interspecific variation in a commonly used measure of innate immunity (total Phenoloxidase or potential PO activity). Two of five species pairs had interspecific differences in proportions of individuals resisting larval *Arrenurus* water mites, only one of five species pairs had species differences in prevalence of larval *Arrenurus* water mites, and another two of five species pairs showed species differences in mean PO activity. Within the two species pairs where species differed in proportion of individuals resisting mites the species with the higher proportion did not have correspondingly higher PO activity levels. Furthermore, the proportion of individuals resisting mites mirrored prevalence of parasitism in only one species pair. There was no interspecific variation in median intensity of mite infestation within any species pair. We conclude that a species’ relative ability to resist particular parasites does not explain interspecific variation in parasitism within species pairs and that neither resistance nor parasitism is reflected by interspecific variation in total PO or potential PO activity.

## Introduction

Explaining interspecific variation in parasitism among related host species has been one of the main goals of evolutionary parasitology [1]. Comparing distantly related species can be difficult because of uncontrolled differences in environmental factors and actual identity and numbers of parasites exploiting the host species. Intriguingly in some taxonomic groups, variation in parasitism can occur among related host species even when ecological variables like habitat use and parasite species exploiting hosts are controlled [2]. In other associations, ecological and evolutionary factors such as geographical range, phylogeny or coevolution can explain differences in parasitism between host species [3], but unexplained variation often remains. One important research gap is the extent of tolerance and resistance between related host species to shared parasites [4]. A first look at this problem would be to compare levels of resistance to real parasites with respect to levels of parasitism and host defences, as done recently by Vogelweith et al. [5].

Evaluating patterns of innate immunity among host species might help predict resistance to parasites and/or interspecific variation in parasitism. In invertebrates, a well-documented defense mechanism and a measure of innate immunity is the encapsulation of foreign substances through the process of melanisation via the Phenoloxidase (PO) cascade [6–10]. Total PO or potential PO activity is one of the proxies to measure the constitutive levels of immune response, i.e. measuring immune levels in the absence of an immune challenge [11]. A host species is expected to have fewer parasites [12] and survive microbial infections [13] more readily with higher levels of PO activity because that species will have greater defences against a broad spectrum of parasites. Even though the activation of phenoloxidase is important in insect immune responses [14] there are at least three general reasons why relations between such proxies of innate immunity and actual parasitism might not be found. First, other proxies of innate or constitutive immunity might be more important to defense including hematocyte density, hemolymph lysozyme activity and nitric oxide levels [11]. Such proxies are important [9,12,15]. Second, the parasites might not be recognized as foreign or might be recognized but not pose much of a cost. Finally, potential PO activity might be more relevant to other physiological functions for a host such as cuticle melanisation (including egg tanning), thermoregulation, crypsis or aposematism [16].

Tests relating PO activity to immunity have often been done at the intraspecific level because variation in PO within species is expected to relate to naturally occurring parasites, but only after controlling for environmental differences between populations [17]. Intriguingly, Iserbyt *et al*. [17] showed that within the female polymorphic damselfly *Nehalennia irene* (Hagen), one female morph has significantly higher total PO activity levels compared to the other female morph. However, these morph-specific differences in total PO activity (hereafter potential PO activity) do not explain variation in parasitism by either gregarines [18] or mites [19]. Additionally, age, size and sexual ornamentation of individuals can be associated with PO activity levels: in *Lestes viridulus* (Rambur), older individuals had less PO activity [20], and in *Hetaerina americana* (Fabricius) the larger individuals with larger wing spots had higher levels of PO activity [21]. In these circumstances, there is considerable variability in PO activity within species, some of which might relate to immunity either directly or indirectly [15].

Although the reliability of PO activity in terms of immune function has recently been questioned [22], PO remains a key enzyme in the production of melanin. Differences in PO activity between closely related species might be important in explaining interspecific variation in immunity, which has been understudied. Host species under higher parasite pressure should invest more in innate immunity than related species with less or no pressure from the same (or similar) parasites. Exploring interspecies variation in immunity and its relation to parasitism will aid in our understanding of the extent to which functional resistance to macroparasites actually does mirror PO activity and, in turn, whether or not either PO activity or a separate measure of functional resistance parallels levels of parasitism, seen between related host species.

**Fig 1:**
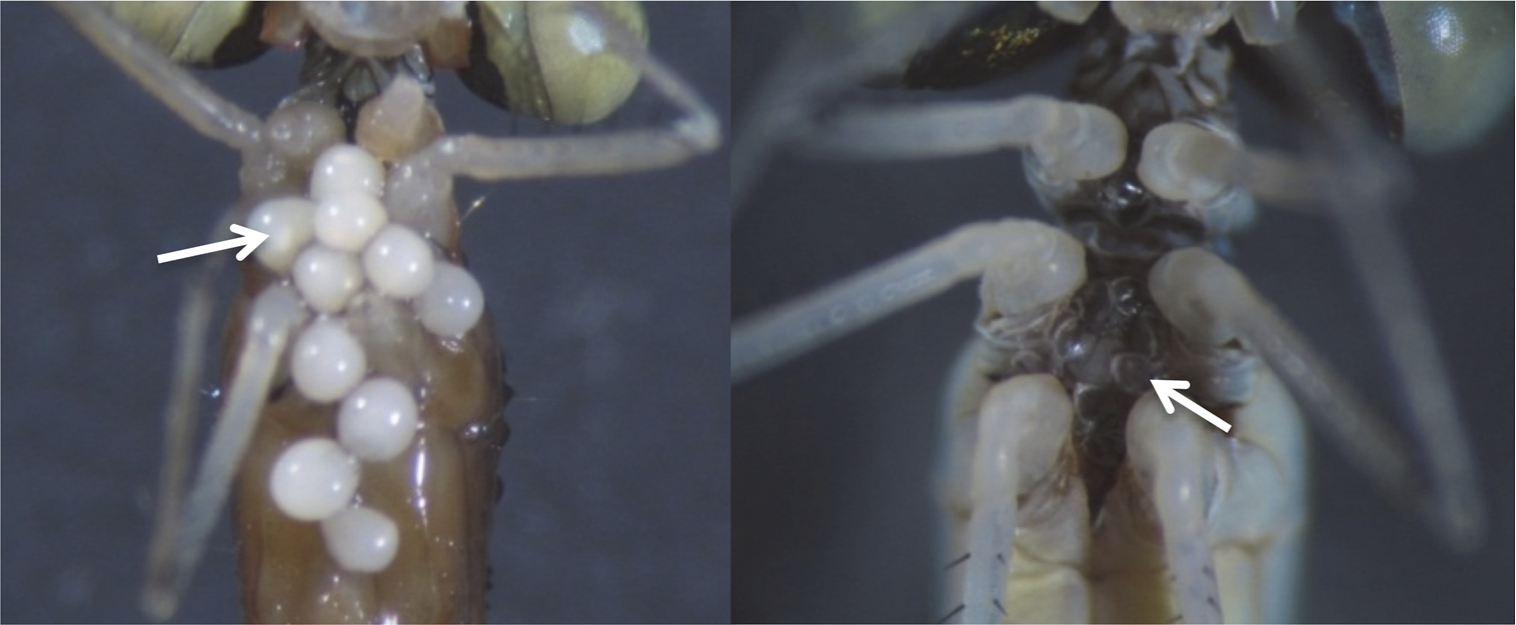
Comparison between live and resisted *Arrenurus* water mites, left image represents engorged live water mites on a *Nehalennia irene* individual, the right picture represents resisted dead water mites on a *Nehalennia gracilis* individual.

The purpose of this study was to determine whether interspecific variation in the proportion of individuals resisting their parasites can explain differences in prevalence and intensity of parasitism for closely related host species and if a constitutive measure of innate immunity (total PO or potential PO activity) can predict interspecific variation in the proportion of individuals resisting their parasites. In order to answer these questions, we use coenagrionid damselfly-*Arrenurus* water mite associations. Damselflies are a unique model hosts to investigate innate immunity and host-parasite interactions for two main reasons. First, resistance to water mites can be easily assessed [23,24]. In damselflies, the immunological response to water mites is melanotic encapsulation of the mites’ feeding tubes or glands responsible for producing feeding tubes [25]. Once the feeding tube has been melanised, the starved water mite remains attached to the host throughout the rest of the host’s adult life [21] (Fig. 1). Second, studies have shown that different but closely related host species have variable levels of both mite parasitism and resistance to same or similar mite species [2,19].

We had two specific objectives. First, we examined interspecific variation in prevalence and intensity of mite parasitism in relation to the proportion of individuals resisting *Arrenurus* water mites in ten species of coenagrionid damselflies (grouped into five species pairs to control for phylogenetic relatedness among host species). Second, we observed whether the magnitude of differences in PO activity mirrored the magnitude of differences in natural resistance to mites and whether either of these measures explained differences in either prevalence or intensity of infestation by mites. We therefore addressed interspecific variation in insect resistance and determined whether this variation is predictive of parasitism levels or can be predicted between related host species based on differences in potential PO activity. To facilitate such comparisons, we studied closely related host species where site and timing of collection had been controlled, and where similar or same ectoparasitic mites were known to attack sibling species of host damselflies.

## Methods

### Host Collecting

Queens University Biological Station granted permission for collection of the insect damselfly species because all were collected on their stations land. The field studies did not involve endangered or protected species. Using aerial sweep nets, we collected individuals from ten Coenagrionidae species, *Argia moesta* (Hagen), *Argia violaceae* (Burmeister), *Enallagma boreale* (Selys), *Enallagma ebrium* (Hagen), *Enallagma signatum* (Hagen), *Enallagma vesperum* (Calvert), *Ischnura posita* (Hagen), *Ischnura verticalis* (Say), *Nehalennia gracilis* (Morse), and *Nehalennia irene* (Hagen), placed in five species pairs, *Argia*, *Enallagma* (*Enallagma*) subgenus, *Enallagma* (*Chromatallagma*) subgenus, *Ischnura*, and *Nehalennia* around the Queens University Biological Station. The four *Enallagma* species are considered in two different species pairs, because the most current phylogeny for this species-rich genus places these four species into two distinct subgenera [26]. Past studies have used these species to discern interspecific variation in parasitism with internal and external parasites [2,19]. Both species within a species pair were collected at the same site and same time to exclude potential site or seasonal effects on parasitism. The *Nehalennia* species pair was collected at Hebert Bog (44°29’54.69”N, 76°24’ 53.66”W) on 7 and 30 June 2011, and 24 June 2012; *E*. (*Chromatallagma*) and *Argia* species pairs were collected at the edge of Lake Opinicon (44°33’56.32”N, 76°19’26.46”W) on 30 June to 3 July, 2010 and from 3 to 10 July, 2010, respectively, and 17 July 2012; the species of the *E*. (*Enallagma*) clade were collected at Barb Marsh (44°31’27.54”N, 76°22’25.89”W) on 25 May 2010, from 7 to 10 June 2010 and on 20 June 2012; *Ischnura* species pair was collected at Stony Swamp (44°30’43.74”N, 76°23’39.32”W) on 4 and 10 July 2011, and on 1 July 1012. There has been little variation in mite prevalence and resistance in consecutive years in these damselfly populations for given species [27]. Collections made in 2010 or 2011 were used to assess host species differences in resistance and levels of parasitism by the same or similar mite species, whereas the samples of 2012 were used to assess species similarities or differences in PO activity, within species pairs.

### Prevalence and intensity of mite infestations and proportion of individuals resisting mites

Immediately upon collection, damselflies were stored in separate vials containing 95% ethanol until processing. Each damselfly was examined with a Zeiss SteREO Discovery.v8 dissecting microscope. Resisted *Arrenurus* water mites were recognized as flat, dark with no separation between the ventral sclerites or dorsal plate in comparison to live mites which were round, red or white (if preserved in ethanol) with ventral sclerites separated by non-scleritized tissue [25] (Fig. 1). Numbers of live and resisted *Arrenurus* water mites were tallied to provide a proportion of dead mites for each species. It is common for closely related host species collected from the same site to share the same *Arrenurus* species [19].

Prevalence of *Arrenurus* parasitism was the proportion of individuals infected with at least one water mite and intensity was the number of water mites per infected individual [28]. Proportion of resisting host individuals was defined as the proportion of infected host individuals with at least one resisted (dead) water mite. We made the assumption that all the resisted mites were killed of melanisation by the host because this has been shown in other damselfly-mite associations, *cf*. [24,29], and because there was rarely high aggregation of parasites in the same body region that would suggest severe competition between the mites and starvation as a cause of mite death.

### Innate immunity (total Phenoloxidase or potential PO activity)

For potential PO activity measures, adult damselflies were collected at the same sites in 2012, as for collections upon which parasitism and resistance analyses were based in 2010 and 2011. In 2012, we collected only hosts that were not infested by water mites to avoid PO already having been mobilized to combat parasitism, and thus not reflecting true innate immunity. All individuals were kept at low temperature immediately after capture to prevent thermo sensitive activation of the PO enzyme. In detail, the damselflies were stored in separate vials and placed into an ice-filled cooler until further processing at the lab [17]. Collected individuals were stored overnight in a fridge at 3°C. After removal from the fridge, each individual was placed in liquid nitrogen for 10 s and placed in -80°C freezer.

The protocol used for the PO assay is as in Stoks *et al*. [30] and Iserbyt *et al*. [17]. In brief, the damselflies were dissected by removing the head, pronotum, wings, legs and abdomen from the excised thorax. The thorax, in an eppendorf tube, was dipped in liquid nitrogen for 10 s and crushed with a hand-held pestle. All dissections were performed on a cooled ice-pack (-20°C) and all necessary products for the PO assay were cooled to 4°C to further reduce enzymatic degradation. Furthermore, all assays were performed within 3 months after sample collection. In order to extract the PO, 300μl of cooled cacodylate buffer (0.01M C_2_H_6_AsNaO_2_–0.005M CaCl_2_) was added to each eppendorf tube containing the crushed thorax. The cell walls of the thorax were removed by centrifugation at 4°C at a speed of 15000rpm for 10 min. From each thoracic extract, 100μl was placed into a well of the 96-well microplate along with 35μl PBS buffer, 5μl α-chymotrypsin and was allowed to react for 5 min. After five minutes, 60μl of L-DOPA (10mmol/L in cacodylate buffer) was added followed by thorough shaking.

Next, the cooled microplate was placed in a spectrophotometer (FLUOstar OPTIMA microplate reader, BMG Labtech). The temperature sensitive reaction proceeded for 30 min at 30°C using an excitation filter of wavelength 485 nm. Twenty cycles were performed with 89-s duration per cycle. All absorbance readings were measured at the beginning of each cycle. Between two readings, the microplate was shaken for 10 s prior to the beginning of a new cycle. Spectral changes occur during this 30-min period due to temperature sensitive nature of this enzymatic reaction. PO activity was scored as the slope of the absorbance-time regression during the linear phase of the reaction. The first five measurements were consistently disregarded because the enzymatic reaction, hence the regression fit, had not yet reached the appropriate linear phase.

As part of a correction for the PO assay, we measured and corrected for thoracic protein content. Total protein content (including the content of the PO enzyme) was expected to rise with individual body size. PO activity was controlled for total protein content, we calculated the PO absolute values of the residuals by regression PO activity against total protein content [17,30]. The protein content was assessed as follows: 5μl of the thorax extract was placed in a new 96-well microplate with 155μl Mili-Q and 40μl Bradfort reagent. The microplate was placed in the spectrophotometer (FLUOstar OPTIMA microplate reader, BMG Labtech) using an absorption filter wavelength of 595. The reaction was run at 30°C for 6 min with continuous shaking. Protein content was then determined by an endpoint reading and, within each plate, compared with a standard curve with known concentrations of Bovine serum albumin (United States Biochemical Corp, Bath—UK). Both physiological parameters (potential PO activity and protein content) were assayed twice to allow replication and to reduce measurement errors. The first and second measurements were typically strongly correlated for potential PO activity (Pearson correlation: r = 0.99; N = 427; P <0.001) and for Protein content (Pearson correlation: r = 0.89; N = 427; P <0.001; see also [17]). The mean of both readings were used for further analyses.

### Statistical analyses

Fisher Exact two-tailed tests were performed to explore for differences in prevalence of infestation and proportion of individuals resisting between the sexes within each species and for species differences within closely related species pairs (sexes separated and sexes pooled as described below). A total of ten Fisher Exact two-tailed tests were run for differences in prevalence and proportion of individuals resisting in each species pair. This test was performed because the sample sizes for certain species pairs (i.e. *Ischnura*) were small [31] and it allowed to keep the statistical analyses consistent for all the species pairs. For differences in median intensity of infestation within species pairs, five separate Mann-Whitney U-test were performed for the five independent species pairs.

To test for differences in potential PO activity between species in the species pairs, separate full factorial design General Linear Mixed Models (GLMMs) were run with PO activity as the response variable and species, sex and thoracic protein content (to control for the size of host [32]) as predictor variables for each of the five species pairs. To determine whether interspecific variation in PO activity explains proportion of resisting individuals and whether either is related to levels of parasitism, we first tested for significant differences among our the variables, separately. All analyses were performed in JMP 10.0.2 (SAS, 2012).

## Results

### Prevalence and intensity of mite infestations and proportion of individuals resisting mites

**Fig 2:**
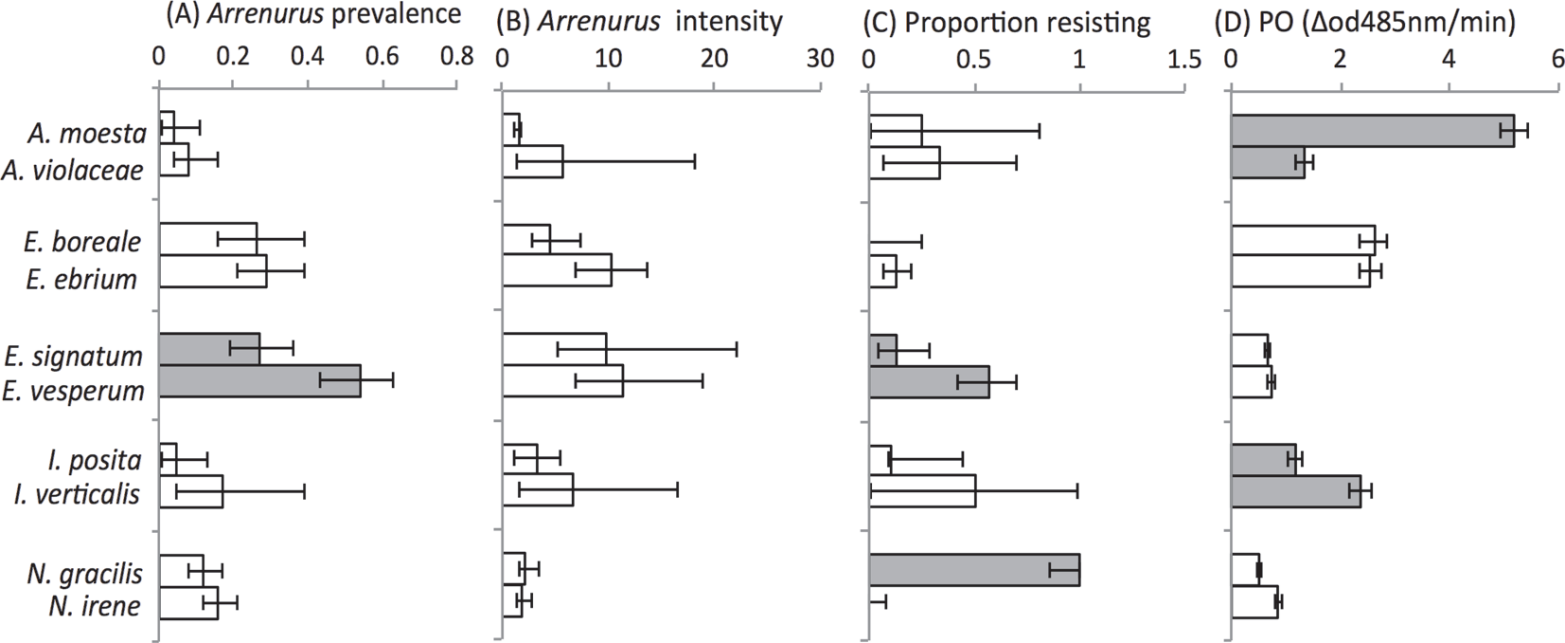
Differences between (A) Prevalence of *Arrenurus* water mites parasitism, (B) intensity of *Arrenurus* water mite parasitism, (C) proportion of *Arrenurus* resisting individuals, and (D) the measurement of PO activity that is activated as an immune response in ten coenagrionid damselfly species grouped by species pair. Prevalence of resisted mites (error bars ±95% Clopper-Pearson confidence intervals) is defined as the percent of the proportion of infected damselflies which resisted at least one water mite parasite. PO activity (error bars ±SE) is based on mean values from the PO assay controlled for by thoracic protein content. Grey bars = significant differences within a species pair.

A total of 1451 damselflies were collected to tabulate infection rates and natural resistance (Table 1). Prevalence of *Arrenurus* parasitism varied from 4% (1–10%, 95% Clopper-Pearson confidence interval) in *A*. *moesta* to 52% (42–62%, 95% Clopper-Pearson CI) in *E*. *vesperum* (Fig. 2A). For infected host individuals, intensity ranged from one water mite per host individual in *A*. *moesta* to 5.7 water mites per infected individual in *E*. *vesperum* (Fig. 2B) and proportion of hosts resisting mites ranged from no host individuals resisting parasites in *E*. (*E*.) *boreale* and *N*. *irene* to all host individuals resisting water mites in *N*. *gracilis* (Fig. 2C). We first tested for significant differences among our variables; the relation between proportion of resisting individuals and total dead mites across species was correlated (Spearman r = 0.77, p = 0.009, see Table 1 for actual values of both measures). Hereafter, we use only the proportion of resisting individuals as our measure of functional resistance (because each individual is only counted once in tabulations).

**Table 1:** Details of *Arrenurus* spp. parasitism, resistance and innate immunity measures in 10 species of damselflies sampled at five sites.

Species pair	Species	Resistance					Innate Immunity		
		Sex	N_1_	N_i_	N_r_	%dead	N_2_	Slope	PO correct
Argia	A. moesta	All	90	4	1	1/5	60	5.18±0.25	1.77±0.17
		M	60	2	0	0/3	30	5.58±0.33	1.94±0.24
		F	30	2	1	1/2	30	4.78±0.37	1.60±0.25
	A. violaceae	All	97	9	3	6/58	24	1.34±0.16	2.75±0.16
		M	61	3	0	0/4	19	1.15±0.13	2.94±0.13
		F	36	6	3	6/52	5	2.07±0.51	2.01±0.51
Enallagma	E. boreale	All	99	23	0	0/145	40	2.61±0.24	1.18±0.14
(E)		M	91	19	0	0/124	34	2.44±0.26	0.94±0.43
		F	8	4	0	0/21	6	3.52±0.43	1.22±0.15
	E. ebrium	All	311	104	13	26/901	30	2.56±0.19	0.78±0.12
		M	288	95	12	18/718	30	2.56±0.19	0.78±0.12
		F	23	9	1	8/83	0	NA	NA
Enallagma	E. signatum	All	120	32	4	5/336	28	0.67±0.06	0.26±0.03
(c)		M	84	23	4	5/256	14	0.53±0.07	0.30±0.03
		F	36	9	0	0/80	14	0.81±0.07	0.22±0.04
	E. vesperum	All	101	53	30	73/580	49	0.74±0.06	0.32±0.04
		M	67	33	18	44/378	49	0.74±0.06	0.32±0.04
		F	34	20	12	29/202	0	NA	NA
Ischnura	I. posita	All	124	10	1	1/11	27	1.18±0.12	0.90±0.09
		M	70	6	1	1/5	16	0.93±0.11	1.09±0.11
		F	54	4	0	0/6	11	1.54±0.21	0.63±0.12
	I. verticalis	All	17	2	1	1/1	59	2.37±0.20	1.21±0.13
		M	10	2	1	1/1	28	1.44±0.17	0.91±0.10
		F	7	0	0	0/0	31	3.22±0.27	1.45±0.22
Nehalennia	N. gracilis	All	208	24	24	51/0	49	0.51±0.04	0.21±0.03
		M	175	21	21	46/0	33	0.45±0.04	0.18±0.03
		F	33	3	3	5/0	16	0.62±0.08	0.29±0.05
	N. irene	All	283	45	0	0/80	60	0.87±0.06	0.37±0.04
		M	240	34	0	0/57	32	0.71±0.05	0.26±0.03
		F	43	11	0	0/23	28	1.05±0.11	0.49±0.07

N_1_ = total sample size used for estimating prevalence and intensity of infection and proportion of resisting individuals by water mites

N_i_ = number of the total number of hosts that were infected

N_r_ = number of total number of infected hosts that resisted one or more mites, %dead = proportion of attached mites being dead and

N_2_ = Total sample size for innate immunity part of the study.

Innate immunity is estimated as phenoloxidase (PO) activity, presented by the slope of the kinetic reaction (Δod485/min) and by PO corrected for protein content by using absolute values of the residuals of PO activity by total protein content regression within each species pair. Mean ± 1 SE is given.

No sex differences in prevalence of *Arrenurus* parasitism were observed within each species, except for *Argia violaceae* (P = 0.048), where males had less mites attached than did females (S1 Table). There was no significant effect of sex in any of the species pairs in explaining variation in median intensity (S1 Table).

Based on the Fisher Exact two-tailed tests for differences between species within species pairs, only *Enallagma* (*Chromatallagma*) had a significant (P<0.001) difference in prevalence of *Arrenurus* parasitism observed. *E*. *vesperum* has a larger proportion of infected individuals (Fig. 1A). There was a highly significant effect of species on proportion of individuals resisting mites in two species pairs (Table 2, Fig. 2C) regardless of whether the sexes were pooled or were separated (S1 Table). In *Enallagma* (*Chromatallagma*), *E*. *vesperum* was more likely to resist water mites than its close relative *E*. *signatum* (p<0.007) and in *Nehalennia*, *N*. *gracilis* was more likely to resist mite parasites than *N*. *irene* (p<0.001). In the other three species pairs, there was no significant difference in proportion of resisting individuals (Table 2). Based on the Mann-Whitney U-tests, intensity of *Arrenurus* parasitism was not statistically different between sibling species in any of the species pairs (Fig 2B; please refer to S2 Table for raw data on measures of parasitism and resistance to water mites.)

**Table 2:** Fisher exact two-tail test results of the differences in prevalence and proportion of individuals resisting water mite parasites and Mann-Whitney U-test for differences in intensity between the species within each damselfly species pair.

Host species pair	Prevalence	Intensity		Proportion of individuals resisting
	P	Z	P	P
Argia	0.254	0.00	1.00	1.000
*Enallagma* (C)	<0.001	1.06	0.290	<**0.001**
*Enallagma* (E)	0.062	1.09	0.277	0.123
Ischnura	0.639	0.25	0.803	0.318
Nehalennia	0.235	0.92	0.360	<0.001

### Innate immunity (total phenoloxidase or potential PO activity)

A total of 427 damselfly individuals were used for the PO and thoracic protein assays (Table 1). PO activity ranged from 0.51 ΔOD485nm/min (±0.04 SE) in *N*. *gracilis* to 5.18 ΔOD485nm/min (±0.25 SE) in *A*. *moesta* (Table 1). Based on the GLMM analyses, in two of the five species pairs, there was significant effect of species on potential PO activity (Fig. 2D). *Argia moesta* had significantly higher PO activity (F_1,76_ = 58.55, p<0.001) than *A*. *violaceae*. Similarly, in *Ischnura*, PO activity was significantly higher (F_1,78_ = 10.55, p = 0.002) in *I*. *verticalis* than in *I*. *posita*. There was no effect of species on potential PO activity in the *Enallagma* (*C*.), *Enallagma* (*E*.) or *Nehalennia* species pairs (Table 3). Sex had a significant on potential PO activity in *Ischnura* (F_1,78_ = 17.48, p<0.001), where males had less potential PO activity than females, but sex had minimal effect on potential PO activity in any of the remaining species pairs (Table 3). We could not test for sex differences in potential PO activity in *Enallagma* (*E*.) and *Enallagma* (*C*.) because no females of *E*. *ebrium* or *E*. *vesperum*, respectively, were caught. The females of *E*. *boreale* and *E*. *signatum* were therefore excluded from the GLMM analyses for these two species pairs (please refer to S3 Table for raw data on PO activity).

**Table 3:** GLMM results of species determining the differences in PO activity within each damselfly species pair.

Host species pair	Effect	df	F	P
Argia	Species	1, 76	58.55	<0.001
	Protein content	1, 76	0.00	0.978
	Sex	1, 76	0.07	0.797
	Species*Protein content	1, 76	0.59	0.445
	Species*Sex	1, 76	2.52	0.117
	Sex*Protein content	1, 76	1.15	0.286
	Species*Protein content*Sex	1, 76	0.57	0.451
Enallagma E	Species	1, 60	0.00	0.995
	Protein content	1, 60	0.58	0.447
	Species*Protein content	1, 60	3.08	0.084
Enallagma C	Species	1, 57	3.20	0.079
	Protein content	1, 57	1.18	0.282
	Species*Protein content	1, 57	1.06	0.308
Ischnura	Species	1, 78	10.55	0.002
	Protein content	1, 78	0.54	0.465
	Sex	1, 78	17.48	<0.001
	Species*Protein content	1, 78	0.28	0.597
	Species*Sex	1, 78	1.99	0.162
	Sex*Protein content	1, 78	0.18	0.669
	Species*Protein content*Sex	1, 78	0.13	0.721
Nehalennia	Species	1, 95	2.56	0.112
	Protein content	1, 95	1.32	0.253
	Sex	1, 95	1.96	0.165
	Species*Protein content	1, 95	0.06	0.813
	Species*Sex	1, 95	1.86	0.176
	Sex*Protein content	1, 95	0.71	0.403
	Species*Protein content*Sex	1, 95	0.01	0.914

GLMM results include the full factorial effect of species, sex and thoracic protein content. Significant differences for the species differences are in bold. Sex was not included in the *Enallagma E* and *Enallagma C* species pairs because no females were collected for those species.

Proportion of resisting individuals was significantly different in *Enallagma* (*C*.) and *Nehalennia* but only *Enallagma* (*C*.) also showed the predicted difference in prevalence of *Arrenurus* parasitism (Fig. 2). Significant differences in potential PO activity did not parallel any of the significant differences in prevalence of parasitism or in proportion of resisting individuals (Fig. 1).

## Discussion

Within host species pairs, the measures observed in this study (prevalence, intensity, proportion of mite-resisting individuals and potential PO activity) did not parallel one another. For example, a species with higher proportion of mite-resisting individuals was not the same species as the one with higher potential PO activity. Within the genera *Argia* and *Ischnura*, one species had significantly higher potential PO activity but proportion of mite-resisting individuals was not different between the two species in each species pair. Interestingly, closely related species often have similar levels of potential PO activity. For example, *Nehalennia* species showed no difference in PO activity but *N*. *gracilis* had proportionally more individuals that resisted mites. Similarly in *Enallagma* (*Chromatallagma*), there was no difference in potential PO activity but *E*. (*C*.) *vesperum* had more mite resisting individuals. Finally, in *Enallagma* (*Enallagma*), there was no significant difference in proportion of mite-resisting individuals or in potential PO activity. Even though yearly environmental factors could have effects when comparing potential PO activity and resistance, such variation is controlled for in the comparison between levels of parasitism and proportions of mite-resisting individuals.

The main interspecific differences documented (*Argia* and *Ischnura* for potential PO activity, and *Enallagma Chromatallagma* and *Nehalennia* for proportion of mite-resisting individuals) were puzzling because there was no obvious pattern across species pairs, among the measures taken. There are possible reasons for the documented singular patterns. In *Argia*, cuticular coloration may explain the differences in potential PO activity, as phenoloxidase is an important enzyme in the formation of cuticular melanin. *Argia moesta* is larger, darker and has higher potential PO activity than *A*. *violacaea*. In mealworm beetles, darker individuals had higher immune capacity than their paler counterparts [33], but in the case of Argia a darker cuticle does not translate into a higher proportion of mite-resisting individuals. In *Ischnura*, differences in potential PO activity may be due to the parasite pressure on the more ubiquitous species *I*. *verticalis*. Host species present at more sites are expected to have higher numbers of interspecific interactions and such species are expected to require more general protection from a greater assortment of parasites, than their less well-distributed counterparts [34]. In the case of *Enallagma* (*C*.), *E*. *vesperum* has higher water mite prevalence and higher proportion of mite-resisting individuals than *E*. *signatum* [35]. These *Arrenurus* species could have co-evolved with *E*. *vesperum* whereby this host species is able to recognize and defend itself at least partially against the parasite. Similarly strong species differences, in resistance to parasites, have been observed in *Nehalennia* sampled from bogs [30] and *Sympetrum* from ephemeral ponds [25]. Specifically, *N*. *gracilis* resists the parasites [35] whereas *N*. *irene* does not. In line with earlier suggestions, *Nehalennia gracilis* may have evolved to recognize parasites because it exists in fragmented populations with limited gene flow [36]. This general hypothesis might also help explain variation in resistance expression within species pairs of *Sympetrum* [24].

Importantly, variation in immune response and resistance has been documented in many studies examining the costs and benefits of resistance to pathogens such as bacteria. In invertebrates, studies demonstrate that genetic heterogeneity influences resistance to pathogens between distinct populations of *Drosophila* [37], *Daphnia* [38], *Bombus* [39] and *Calopteryx* [40]. Additionally, another study demonstrates that two sympatric species of totricid moths respond differently to parasitoids [5]. Our study differs from the earlier studies, but is similar to the moth study, because we include variation in resistance between different host species collected from the same site and not different populations of a species from different sites. Additionally, resistance in these studies is not as easily observed as for the damselfly-water mite host-parasite system because resisted (dead) water mites remain on the host damselfly. Recently, a study compared immune responses to nylon inserts in two differentially resisting lestid damselflies [23]. The authors found that *Lestes forcipatus* (Rambur) which resists its parasites more readily melanises the inserts more than *Lestes disjunctus* (Selys), a species with lower resistance. In the case of lestid damselflies, it seems that a species resistance and its induced immune responses are linked when the specificity of the challenge is controlled for. In our study, parasites might have evolved evasion of recognition by some host species’ immune systems because of the co-evolutionary history between certain damselfly hosts- *Arrenurus* parasites.

As mentioned, there are caveats for using PO activity as an appropriate measure of innate immunity in insects and our study further emphasizes this point. Gonzalez-Santoyo *et al*. [22] reviewed the success of PO activity in insect immunity; based on the literature, 11 of 23 papers that reported PO activity in terms of innate immunity found a positive relationship between PO activity and successful pathogen defence. Of the remaining 12 papers, half found a negative relationship and the other six found no relationship. The main explanation for this variability is suggested to be the high costs of this defence mechanism and its dependence on resources acquired in the past by the host. Recently, Moreno *et al*. [9] tested the range and efficacy of currently used proxies of immune response including PO activity. Their major issue with PO activity was that with long term storage, even in -70°C temperature, PO activity could be degraded or spontaneously activated. There is no question that PO activity plays a role in insect innate immunity, but it is very species specific. There is a debate about the relevance of PO activity as a proxy for innate immunity because the PO cascade is also used for different functions such as cuticular melanisation, wound healing and egg tanning in females [41,42]. Variation in storage was controlled for in this study (i.e. all the individuals were treated the same way; first held overnight in a fridge at 4°C, frozen in liquid nitrogen the following day and all lab analyses were performed within six months after capture). However, subtle differences between related host species in traits like importance of PO for cuticular melanisation remain unknown.

In conclusion, we found that resistance measured as proportion of mite-resisting individuals among species does not reflect prevalence or intensity of water mite parasitism and neither measure relates to interspecific variation in potential PO activity between related species. In only one case, *Enallagma* (*Chromatallagma*), proportion of mite-resisting individuals reflects prevalence of water mite parasitism. Even though melanisation has a documented immune function in damselflies, investment in PO is likely to be traded off with other uses such as cuticle formation (for blackflies [43]). As Gonzalez *et al*. [22] suggest, our knowledge of ecological immunology is ever increasing, but we do not yet have the full understanding on PO activity. Gross interspecies differences in PO among related species are not often studied, especially in relation to functional immunity. Other innate immunity proxies (i.e. hematocyte counts, nitric oxide and hemolymph lysozyme activity) may be better predictors of resistance to parasites. We encourage future studies of other invertebrate taxa to make investigations into other factors such as host recognition, and not rely on one or more measures of innate immunity in isolation, as is often the case. Such studies will help identify some of the constraints on immune system evolution and function.

## Supporting Information

S1 Table Fisher exact two-tailed tests of the differences in prevalence of water mite parasitism, proportion of individuals resisting water mite parasites and Mann-Whitney U-test for differences in water mite intensity between sexes within ten Coenagrionidae species. (DOCX)

S2 Table Raw data on measures of parasitism and resistance in ten Coenagrionidae species to *Arrenurus* water mites. (XLSX)

S3 Table Raw data on PO activity in ten coenagrionid species. (XLS)
