# Supplementary material for: Differential Water Mite Parasitism, Phenoloxidase Activity, and Resistance to Mites Are Unrelated across Pairs of Related Damselfly Species
Source: PLoS One. 2015 Feb 6;10(2):e0115539. doi: 10.1371/journal.pone.0115539 (PMC4319886; doi:10.1371/journal.pone.0115539)
Supplement: S1 Table — (DOCX) [file pone.0115539.s001.docx]

Appendix

Table S1: Fisher exact two-tailed tests of the differences in prevalence of water mite parasitism, proportion of individuals resisting water mite parasites and Mann-Whitney U-test for differences in water mite intensity between sexes within ten Coenagrionidae species.

| Species | Prevalence | Intensity | | Proportion of mite resisting individuals |
| --- | --- | --- | --- | --- |
|  | P | Z | P | P |
| *Argia moesta* | 0.598 | 0.00 | 0.333 | 1.000 |
| *Argia violaceae* | 0.073 | 0.27 | 0.048 | 0.787 |
| *Enallagma boreale* | 0.082 | 0.21 | 1.000 | 0.806 |
| *Enallagma ebrium* | 0.647 | 0.44 | 1.000 | 0.659 |
| *Enallagma signatum* | 0.826 | 1.70 | 0.315 | 0.089 |
| *Enallagma vesperum* | 0.404 | 0.43 | 0.490 | 0.671 |
| *Ischnura posita* | 1.000 | 0.43 | 1.000 | 0.669 |
| *Ischnura verticalis* | 0.485 | NA | 1.000 | NA |
| *Nehalennia gracilis* | 0.774 | 0.29 | 1.000 | 0.774 |
| *Nehalennia irene* | 0.168 | 0.77 | 1.000 | 0.441 |
